# Partial response of donafenib as the third-line therapy in metastatic colon cancer: A case report

**DOI:** 10.1097/MD.0000000000027204

**Published:** 2021-09-17

**Authors:** Yang Yang, Hong Zhu, Qiu Li

**Affiliations:** Department of Medical Oncology, Cancer Center, West China Hospital, Sichuan University, No. 37, GuoXue Xiang, Chengdu, Sichuan, China.

**Keywords:** case report, colorectal cancer, donafenib, partial response, third-line therapy

## Abstract

**Rationale:**

Colorectal cancer (CRC) is a digestive tumor with high morbidity and mortality rates. After second-line treatment failure, third-line treatment options are limited, and the objective response rate is low. These patients are expected to have a short survival time. Therefore, it is very important to explore safer and more effective treatment options for patients with advanced colorectal cancer. Donafenib is a new type of tyrosine kinase inhibitor developed independently in China. Its effectiveness and safety as a first-line treatment for patients with advanced hepatocellular carcinoma in China have been verified.

**Patient concerns:**

The patient was a 60-year-old Asian man who presented with sudden lower abdominal pain, vomiting, anal exhaustion, and poor defecation, without an apparent cause. He had no history of type 2 diabetes, hypertension, or other relevant past illnesses.

**Diagnosis:**

Metastatic colon cancer (stage IV).

**Interventions:**

mFOLFOX6 chemotherapy was administered in 15 cycles as first-line therapy. FOLFIRI chemotherapy was administered in 8 cycles as second-line therapy. Donafenib was administered as third-line therapy.

**Outcomes:**

The patient achieved partial response. No serious adverse events (grades III–IV) occurred.

**Lessons:**

This case report provides clinicians with a safe and effective option for donafenib as a later-line treatment option for patients with metastatic colorectal cancer to improve their overall survival and quality of life.

## 1 Introduction

Colorectal cancer (CRC) is the fourth leading cause of tumor-related death all over the world. Local treatment methods are mainly surgery and radiotherapy.^[1]^ However, owing to the high recurrence and metastasis rates, systemic treatment is essential in prolonging patient survival. Currently, the objective response rate (ORR) of first-line treatment of CRC is as high as 50% to 70%, while the ORR of second-line treatment is lower, approximately 10% to 30%.^[2]^ After second-line treatment failure, the ORR is less than 10%. Available third-line treatment options are limited. According to the National Comprehensive Cancer Network guidelines, there are only 2 standard regimens, regorafenib and TAS-102, which are third-line treatment option. In addition, fruquintinib is a recommended standard third-line treatment regimen in China.^[3]^ Donafenib is a new tyrosine kinase inhibitor, a targeted drug developed independently in China. Following the ZGDH3 study, it has been recommended as the standard first-line treatment for hepatocellular carcinoma.^[4]^ However, the therapeutic effect of donafenib on patients with CRC remains unknown. The identification of effective third-line drugs for patients with second-line treatment failure is the key for survival time prolongation and quality of life improvement for such patients.

This report describes the case of a patient with metastatic colorectal cancer (mCRC) who had second-line treatment failure and was then treated with donafenib, a third-line drug; during the third-line therapy, he achieved partial response (PR). This report aims to widen the drug choice for doctors who observe second-line treatment failure while managing their patients with mCRC.

## 2 Case presentation

**Figure 1:**
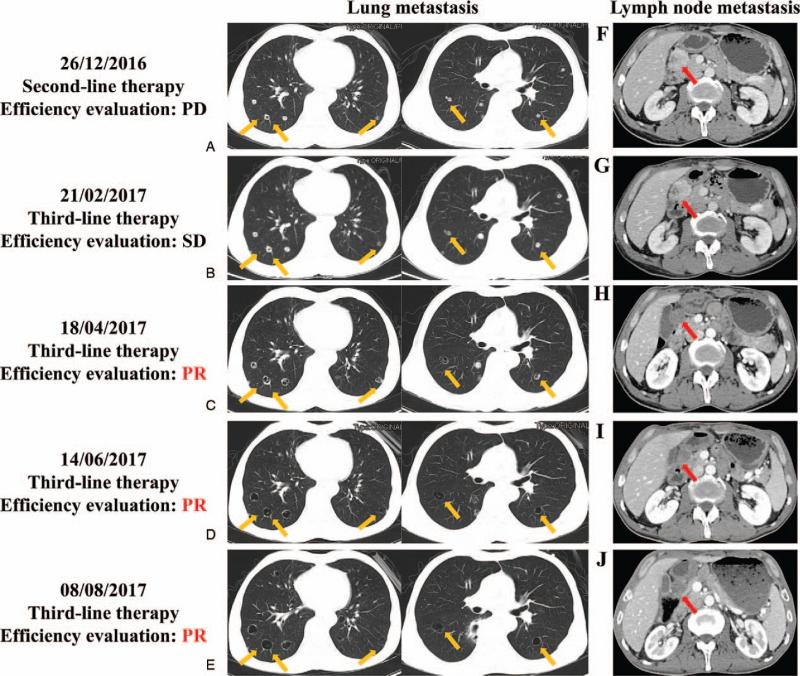
Abdominal and chest enhanced computed tomographic images. (A, B, C, D, E) Lung metastatic nodules (orange arrowhead). (F, G, H, I, J) Lymph nodules in the gastrocolic ligament area (red arrow).

The patient was a 60-year-old Asian man. On April 14, 2014, the patient presented with sudden lower abdominal pain, vomiting, anal exhaustion, and poor defecation, without any apparent cause. He had no history of type 2 diabetes, hypertension, or other relevant past illnesses. A plain film of the abdomen showed incomplete intestinal obstruction. Colonoscopy revealed a right transverse colon neoplasm. Palliative resection surgery for transverse colon cancer was performed in May 2014. Immunohistochemical analysis revealed positive expression of CEA, CK19, CK20, villin, p53, and Ki67, and negative expression of HER-2, CgA, and Syn. Following these findings, a pathological diagnosis of moderate to poorly differentiated stage IV adenocarcinoma of the right transverse colon was made. On June 3, 2014, postoperative chest and abdominal computed tomography (CT) showed that the anastomotic wall of the residual colon cancer was slightly thicker, with a 2-cm superior mass (diameter of 2 cm) invading the duodenum. As first-line treatment, 15 cycles of mFOLFOX6 chemotherapy (d1: oxaliplatin 130 mg, leucovorin 600 mg, fluorouracil 600 mg iv, fluorouracil 1800 mg; d1–2 civ23h q2w) were administered between June 4, 2014, and December 27, 2014. The patient was followed up regularly until the disease was stable. A repeat chest and abdominal CT performed on November 21, 2015, showed an enlargement of the lesion above the anastomosis. Three cycles of mFOLFOX6 chemotherapy regimen were administered between December 16, 2015, and February 24, 2016. On March 25, 2016, CT revealed increased and enlarged lung nodules, suggestive of lung metastasis – this was considered as disease progression. Therefore, second-line treatment using FOLFIRI chemotherapy regimen (d1: irinotecan 280 mg, leucovorin 600 mg, fluorouracil 600 mg iv, fluorouracil 1800 mg; d1–2: civ23h q2w) was administered in 8 cycles between March 29, 2016, and November 28, 2016. On December 26, 2016, CT revealed enlarged lung metastatic nodules (compared to the previous CT findings) and new lung metastases (Fig. 1A, F). During second-line therapy, the patient became very thin. Following disease progression, third-line treatment with oral donafenib (300 mg bid) was administered continuously between December 28, 2016, and August 8, 2017. A repeat CT was performed on February 21, 2017 (Fig. 1B, G), April 18, 2017 (Fig. 1C, H), June 14, 2017 (Fig. 1D, I), and August 8, 2017 (Fig. 1E, J): gradual shrinkage of the lung metastatic nodules with some of them forming cavities was observed. The diameter of the peritoneal mass also decreased while showing signs of liquefaction and necrosis. This was considered as PR. The imaging effect evaluation was performed using the Response Evaluation Criteria in Solid Tumors version 1.1.^[5]^ Evaluation of all adverse events was based on the Common Terminology Criteria for Adverse Events version 5.0.^[6]^ On August 28, 2017, the patient developed constipation (degree II) and decreased appetite. Therefore, donafenib was discontinued. The patient experienced adverse events of donafenib such as bone marrow suppression (grade II reduction in white blood cells and grade I reduction in platelets), diarrhea (grade II), and low serum potassium ion concentration (degree II). However, he did not develop severe side effects (grade III or higher). The patient died on March 26, 2018. The efficacy of donafenib as a third-line treatment was PR, and progression-free survival was at least 8 months.

## 3 Discussion

Second-line treatment of CRC is inefficient, and the expected survival time of patients with CRC is short.^[7]^ Herein, we reported the case of a 60-year-old man who achieved PR with donafenib, which was used as a third-line treatment drug for mCRC. No serious adverse events were observed.

Donafenib simultaneously inhibits the activity of multiple receptor tyrosine kinases, such as VEGFR and PDGFR. To inhibit tumor cell proliferation, it also inhibits various RAF kinases directly and inhibits the downstream RAF/MEK/ERK signal transduction pathways.^[8]^ In the ZGDH3 study on advanced hepatocellular carcinoma,^[4]^ overall survival of the donafenib group was better than that of the sorafenib group. Median overall survival (mOS) of the donafenib and sorafenib group was 12.1 months and 10.3 months, respectively – a difference of 1.8 months. The risk of patients in the donafenib group was 17% lower than that in the sorafenib group. Regarding safety, the safety of donafenib was significantly lower than that of sorafenib, as seen by the serious adverse reactions and adverse events that led to donafenib dose reduction or suspension. Donafenib has been reported to have a better survival benefit and safety than sorafenib in the treatment of advanced hepatocellular carcinoma worldwide: this was observed in the first large-scale phase III clinical trial of single-drug and head-to-head comparison. Therefore, donafenib has a great potential for tumor treatment.

Treatment outcomes of cetuximab and bevacizumab used as first and second-line treatment options, respectively, are very impressive. In the CALGB 80405 study, a combination of cetuximab and chemotherapy achieved an overall survival of 30.0 months, and a mOS of 29.0 months was achieved with bevacizumab as first-line treatment for RAS wild-type advanced or metastatic CRC.^[9]^ In the BECOME study, a mOS of 25.7 months was achieved with a combination of bevacizumab and chemotherapy, compared to 20.5 months with chemotherapy alone for mCRC.^[10]^ Since our patient had a poor general state, he refused to undergo RAS/RAF testing to evaluate the efficacy of cetuximab/bevacizumab treatment. The National Comprehensive Cancer Network guidelines recommend the multitarget drug, regorafenib, as third-line treatment for mCRC. This illustrates the potential of multitarget drugs for CRC treatment. However, our patient could not afford it owing to financial constraints. In addition, he accepted first-line and second-line therapy and needed to accept other third-line therapy regimens. Therefore, we recommended donafenib to our patient. The dosage was determined based on the phase 1/2 study of donafenib-treated mCRC (NCT02489916). We observed a good treatment response and good safety.

There were shortcomings in the management of this patient: Donafenib has not been verified in large-scale clinical trials because of its clear effectiveness and safety in CRC treatment. The patient did not receive standard targeted therapies such as cetuximab/bevacizumab/regorafenib because of financial constraints. After second-line treatment, the patient became very thin and malnourished. Moreover, donafenib was discontinued after he presented with side effects, constipation (degree II), and decreased appetite.

In conclusion, this case report shows the good efficacy and safety of donafenib as third-line treatment of mCRC. Nevertheless, further research is warranted.

## Author contributions

Yang Yang performed the data acquisition and manuscript preparation. Hong Zhu and Qiu Li performed the radiological analysis of CT images and manuscript conception.

**Investigation:** Hong Zhu.

**Writing – original draft:** Yang Yang.

**Writing – review & editing:** Hong Zhu, Qiu Li.
